# The Influence of Alcohol Consumption on Intestinal Nutrient Absorption: A Comprehensive Review

**DOI:** 10.3390/nu15071571

**Published:** 2023-03-24

**Authors:** Molly Butts, Vijaya Lakshmi Sundaram, Usha Murughiyan, Alip Borthakur, Soudamani Singh

**Affiliations:** Department of Clinical and Translational Sciences, Marshall University, Huntington, WV 25755, USA; butts15@marshall.edu (M.B.); borthakur@marshall.edu (A.B.)

**Keywords:** alcohol, ethanol, intestine, nutrient absorption, brush border membrane

## Abstract

Chronic alcohol use has been attributed to the development of malnutrition. This is in part due to the inhibitory effect of ethanol on the absorption of vital nutrients, including glucose, amino acids, lipids, water, vitamins, and minerals within the small intestine. Recent advances in research, along with new cutting-edge technologies, have advanced our understanding of the mechanism of ethanol’s effect on intestinal nutrient absorption at the brush border membrane (BBM) of the small intestine. However, further studies are needed to delineate how ethanol consumption could have an impact on altered nutrient absorption under various disease conditions. Current research has elucidated the relationship of alcohol consumption on glucose, glutamine, vitamins B1 (thiamine), B2 (riboflavin), B9 (folate), C (ascorbic acid), selenium, iron, and zinc absorption within the small intestine. We conducted systematic computerized searches in PubMed using the following keywords: (1) “Alcohol effects on nutrient transport”; (2) “Alcohol mediated malabsorption of nutrients”; (3) “Alcohol effects on small intestinal nutrient transport”; and (4) “Alcohol mediated malabsorption of nutrients in small intestine”. We included the relevant studies in this review. The main objective of this review is to marshal and analyze previously published research articles and discuss, in-depth, the understanding of ethanol’s effect in modulating absorption of vital macro and micronutrients in health and disease conditions. This could ultimately provide great insights in the development of new therapeutic strategies to combat malnutrition associated with alcohol consumption.

## 1. Introduction

Among Americans, alcohol consumption is very common practice according to the National Institute on Alcohol Abuse and Alcoholism (NIAAA). However, alcohol consumption, even at low to moderate dosages, could affect human physiology in a multitude of organ systems, including the small intestine [1,2]. The main purpose of the small intestine is to absorb digested nutrients essential for homeostasis [3]. These nutrients are passively absorbed or actively transported through the brush border membrane (BBM) of small intestinal epithelial cells called enterocytes [4]. There are multiple mechanisms by which absorption of nutrients in the small intestine may occur. For example, small molecules, such as glycerol, gases (O_2_ and CO_2_), and products of dietary lipids digestion, becomes absorbed via simple diffusion. Some molecules, such as dietary fructose, enter the enterocyte via a facilitated diffusion process mediated by the GLUT-5 transporter. On the other hand, glutamine (B0AT1/SLCA19) and glucose (SGLT1/SLC5A1) are co-transported, along with sodium involving an active transport against a concentration gradient. The energy for this process is provided by the Na-K-ATPase pump in the basolateral membrane of the enterocyte [3,4,5].

The impact of alcohol consumption on nutrient absorption along the small intestine have been extensively studied [1,6,7,8,9,10,11,12,13,14,15,16,17,18,19,20,21,22,23,24,25,26]. In this review, we have attempted to update recent research regarding the mechanisms of alcohol’s effect on nutrient absorption along the small intestine and its alterations in disease conditions. Understanding the underlying mechanisms of alcohol in the alteration of nutrient absorption is vital, as elucidating these mechanisms can lead to a better understanding of alcohol-dependent malnutrition, as well as other nutrition-related disease states.

## 2. Alcohol Use

As per the NIAAA, more than 50% of Americans normally consume alcohol. Statistically, this is important because chronic alcohol consumption can lead to heart disease, high blood pressure, liver disease, and gastrointestinal complications [27,28,29,30,31]. Furthermore, consumption of alcoholic beverages, such as 12 ounces of beer, eight to nine ounces of malt liquor, five ounces of wine, or one and a half ounces of liquor consumed can increase the risk of cancer of the mouth, throat, esophagus, breast, liver, and colon [27,29,30,31]. Many studies focus on heavy drinking, which the NIAAA defines as four alcoholic beverages for women or five for men in two hours, which results in a blood alcohol content (BAC) of 0.08 g/dL. Five or more occurrences of such episodes within one month is considered as binge drinking. According to NIAAA research surveys, the majority of Americans are exposed to lower blood alcohol levels than are heavy alcohol abusers [28]. A single standard alcoholic beverage per day for females and two for males, defined as moderate alcohol consumption, is a rational measure of ethanol to investigate [27,28]. Recent studies have shown that even smaller dosages of ethanol can have significant effects on human physiology [1], nutrient absorption, and can even affect nutrient transporters along the small intestine [32,33].

### The Effect of Alcohol Consumption on the Gastrointestinal Tract

Alcohol, commonly known as ethanol, typically enters the human body along the GI tract. As ethanol travels from the mouth and down the esophagus to the stomach, it increases the risk of mouth, esophageal, and gastric cancer [27,29,30,31]. In the stomach, ethanol undergoes first pass metabolism through the primary ethanol enzymatic reaction via alcohol dehydrogenase 1 and 3 [34]. After first pass metabolism, ethanol is assimilated primarily in the upper small intestine [35]. Due to its amphiphilic nature, ethanol’s absorption occurs via passive diffusion through the plasma membrane of intestinal epithelial cells called enterocytes [36,37]. Several variables influence the rate of ethanol absorption, including, but not limited to, ethanol dosage, amount of ingested food, gastric emptying rate, ethanol concentration, intestinal motility, intestinal wall permeability, and blood flow [34,38]. While, at this point, ethanol is distributed evenly throughout the body, it nonetheless continues to interact with the rest of the GI tract [35]. Ethanol is distributed to the distal small intestine and colon through the mesenteric vasculature. Additionally, it affects the tissues of the lower GI tract through the basolateral membrane (BLM) of enterocytes [39,40,41]. Ethanol’s effect on the BLM has been detailed in several studies [1,30,41,42], with the most convincing study showing luminal changes in the terminal small intestine following intraperitoneal injections of ethanol in mice [41]. A recent study on mouse morphology demonstrated that the small intestine in alcohol-fed mice had shown significant heterogeneity of the villus cell surface and crypts cell deformation [43]. Alcohol studies in animals and humans reported that the tip of the villi is blunted along with the inflammatory cell infiltration and hemorrhage in the lamina propria [23,43,44,45,46].

**Figure 1:**
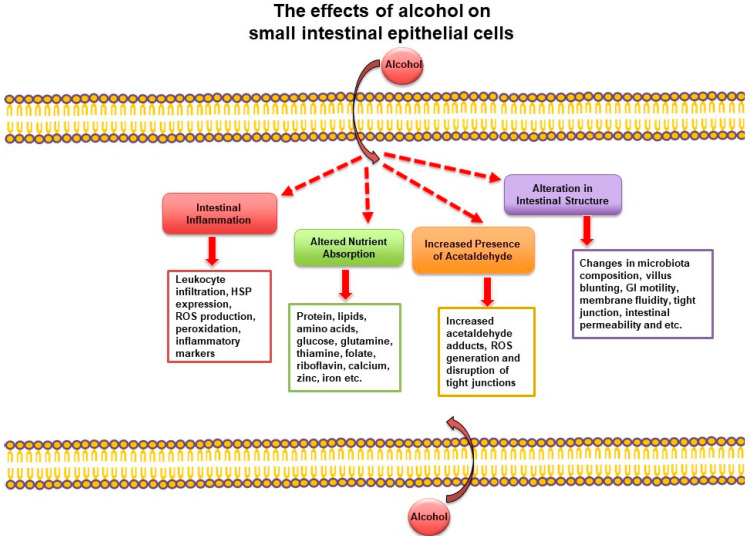
General effects of alcohol consumption on the small intestine. Adapted with permission from Butts M.R. [32]. GI, gastrointestinal; HSP, heat shock protein; ROS, reactive oxygen species.

Patients who consumed alcohol showed more disruption of the gastric mucosal barrier and increased mucosal permeability to immune cells. As ethanol interacts with the GI tract, numerous cellular and structural changes occur (Figure 1). Ethanol has been shown to alter the contractile proteins and vagal functions associated with the intestine, which can alter bowel motility [24]. Acute and chronic dosages of ethanol have been shown to affect the intestinal mucosa by reducing villus height, causing villus blunting, as well as other cellular structural changes [47,48]. Ethanol also affects the composition of the microbiota and causes bacterial overgrowth in the intestine [49,50,51,52,53]. At the cellular level, ethanol alters membrane dynamics [36,37,54], modifies cellular junction proteins [40,55,56,57,58], and causes a variety of subcellular signaling and immune responses [1,41,59]. Ethanol can also alter cellular plasma membrane dynamics by passively inserting into the plasma membrane. In as little as 400 nanoseconds, ethanol can increase the fluidity of the plasma membrane while decreasing bilayer thickness, as shown in computer simulations [36,37] and in Oenococcus oeni cells [54]. In addition to increases in membrane fluidity, ethanol also increases intestinal permeability, especially in the colon [60]. The increased intestinal permeability is due, in part, to the removal of integral membrane proteins, such as ZO-1 and occludin in the tight junction in response to ethanol [40,55,56,57,58]. Furthermore, following treatment with ethanol, both cellular stress responses, such as the induction of heat shock protein 70, and immune responses, such as leukocyte infiltration and histamine release, were altered in the small intestine [1,41,59]. Increases in lipid peroxidation, reduction in antioxidants, and increases in oxidative stress have also been shown to occur in response to ethanol [61,62,63]. Overall, exposure to ethanol clearly alters intestinal homeostasis, including nutrient absorption [1]. Alcohol consumption also affects the digestion of nutrients along the GI tract. Several studies have shown that alcohol affects enzymatic digestion by inhibiting BBM peptidases [1,64]. While the digestion of nutrients plays a significant initial role in the overall absorption of nutrients, our focus in this review is confined to the effect of ethanol on nutrient absorption at the BBM of the small intestine.

## 3. The Impact of Ethanol on Intestinal Nutrient Absorption

This segment of the article will highlight the association between alcohol consumption and altered nutrient absorption. The research began on ethanol’s effect on nutrient absorption when a link between Wernicke Korsakoff syndrome and vitamin B1 deficiency was made (discussed in more detail in the next section). Ethanol’s impact on the BBM vitamin B1 transporter was further studied, which kindled interests in investigating the effects of alcohol consumption on intestinal nutrient absorption [11]. Various studies on the effects of alcohol consumption on the absorption of macronutrients, including water [15,17], carbohydrates, such as glucose [7,10,14,17,65,66,67,68,69,70,71,72,73,74] and xylose, lipids [18,75,76,77,78,79], peptides [78,79], and amino acids, such as glutamine [33], leucine [72,80,81,82], and glycine, [70] have been reported (Table 1). Furthermore, alcohol consumption affects the absorption of micronutrients, including water-soluble vitamins, such as vitamin B1 [11,83,84,85,86,87,88,89], B2 [90], B6 [1]), B9 [8,16,91,92,93,94], B12 [13], and C [95]. Additionally, the absorption of fat-soluble vitamins is affected, including vitamins A, D, E, and K [1,13] and minerals, including calcium [9,96], zinc [97,98,99], iron [100,101], magnesium [102], and selenium [103,104]. Nutrients not included in Table 2 that are decreased in response to a heavy dosage of ethanol include phenylalanine, alanine, methionine, and valine [6], (Table 2). These studies were conducted using a wide variety of concentrations of ethanol, administration techniques, and model systems. In this review, we will focus on the effect of ethanol on intestinal nutrient absorption beginning with vitamin B1.

### 3.1. The Effect of Ethanol on Micronutrient Absorption

#### 3.1.1. Vitamin B1 Absorption

Ethanol’s impact on intestinal thiamine, vitamin B1, absorption has been extensively studied. Vitamin B1 deficiency is directly linked to Wernicke-Korsakoff syndrome and associated brain atrophy [113,114]. Initially, Hoyumpa and colleagues utilized everted rodent jejunal segments to link vitamin B1 deficiency in chronic alcoholics to an inhibition of the active transport of low concentrations of vitamin B1 [11,22,83,84]. The passive transport of vitamin B1 was unchanged. Ethanol’s inhibitory mechanism on the vitamin B1 transporter, upon exposure to heavy dosages of ethanol at the BBM of the small intestine in rats, was due to a decrease in the maximal rate of transport (Vmax) with no change in the affinity of the transporter (1/K_m_) for ethanol [87]. Further investigations showed no significant difference between acute or chronic alcohol exposure in the jejunum of 12 human males [86].

Further studies at the molecular level have shown that, at the BBM and BLM of the rat jejunum, a four-week administration of heavy ethanol reduced the mRNA and protein expression of the vitamin B1 transporter-1 (SLC19A2). Ethanol treatment had no influence on the vitamin B1 transporter-2 (SLC19A3). Ethanol’s inhibitory action was also shown at the level of the vitamin B1 transporter’s transcriptional promoter activity in human intestinal epithelial HuTu-80 cells (SLC19A2–3) [89]. Further studies even demonstrated differences in ethanol’s effect on vitamin B1 absorption based on the type of alcohol consumed. Both wine and ethanol produced an inhibition in vitamin B1 absorption following an acute 12% *v*/*v* binge dosage of ethanol. However, in a longer 21-day treatment, only ethanol caused a decrease in vitamin B1 absorption, whereas wine did not [88]. Clearly, further research into nutritional absorption studies involving different types of alcohol is necessary to fully comprehend ethanol’s underlying impact on intestinal nutrient absorption.

#### 3.1.2. Vitamin B2 Absorption

Ethanol’s impact on intestinal vitamin B2 absorption was not discussed in previous reviews because the link between ethanol and the essential dietary coenzyme vitamin B2, riboflavin, was not described until 2013. Subramanian and colleagues demonstrated that long-term exposure to ethanol via the Lieber DeCarli ethanol diet inhibits intestinal vitamin B2 absorption (SLC52A1 and SLC52A3). This study utilized male Wistar rats that were administered ethanol over four weeks. Then, vitamin B2 absorption at both the BBM and BLM of the small intestine was investigated. It has also been shown that vitamin B2 absorption was inhibited in the colon and the kidney. At the molecular level, ethanol decreased the protein expression of the vitamin B2 transporter and heteronuclear RNA (hn RNA) levels, which measures transcription rate. Future studies are warranted to better extend the knowledge regarding ethanol’s inhibitory impact on vitamin B2 absorption and transporter protein expression [90].

#### 3.1.3. Vitamin B7 (Biotin) Absorption

There have been very few studies that are pertinent to ethanol exposure on intestinal vitamin B7 absorption. Vitamin B7 in mammals is mostly acquired from exogenous sources (dietary and bacterial sources) through intestinal absorption, since most mammals cannot synthesize vitamin B7 endogenously. Although various biotin transporters exist, SLC5A6 (sodium-dependent multivitamin transporter, SMVT) is an intestinal-specific biotin transporter that transports biotin in the intestine [115,116]. Studies conducted by Subramanya et. al demonstrated that biotin uptake was significantly reduced during chronic alcohol exposure in the small and large intestines, and the reduction is due to a significant decrease in the transcription of the SLC5A6 transporter [107]. It has also been demonstrated that chronic alcohol exposure inhibits small intestinal and colonic biotin uptake and SMVT expression in human differentiated enteroid and colonoid monolayers and in Caco-2 cells [108]. The reduction of SMVT expression was attributed to significant downregulation of the expression of the nuclear factor KLF-4 (needed for SLC5A6 promoter activity), as well as via an epigenetic mechanism involving altered methylation of the potential CpG island in the SLCA56 promoter, secondary to alcohol-mediated alteration of histone remodeling via acetylation/deacetylation (histone modifications) [108].

#### 3.1.4. Vitamin B9 Absorption

Vitamin B9 (folate) deficiency in response to ethanol exposure has been extensively studied due to vitamin B9’s known importance during pregnancy as early as the 1970s [16]. In a prior study, it was demonstrated that ethanol did not affect intestinal vitamin B9 absorption [16]. The above finding was recapitulated in another study using wine [88]. However, current research has demonstrated that ethanol does directly affect intestinal vitamin B9 absorption specifically by affecting the activity of intestinal vitamin B9 carriers (SLC46A1; SLC19A1) and receptor [94,109].

In a previous study using Wistar rats fed with 1 g/kg ethanol for three months, investigators described the mechanism of ethanol’s impact on intestinal vitamin B9 absorption [109]. These studies revealed that there was a decrease in the maximal rate of uptake of the transporter (Vmax) and an increase in the affinity of the transporter for its substrate (Km). Ethanol’s inhibitory action was observed throughout the crypt to villus axis of the small intestine, which highlighted a consistent response throughout the intestinal architecture. Investigators have shown the inhibition of vitamin B9 absorption to be partially due to altered localization of the transporter in the BBM [109]. Moreover, the effect of ethanol on vitamin B9 absorption was reversed with a two-month reprieve from ethanol, suggesting that ethanol effects on intestinal vitamin B9 absorption are reversible [94]. Further, molecular studies have described the inhibition of SP1 transcription factor binding at the vitamin B9 carrier promoter regions after ethanol exposure. However, the intracellular mechanism required further investigation [94]. The current literature showed that one of the primary enzymes responsible for enabling intestinal vitamin B9 transport—folylpolyglutamate hydrolase—was significantly downregulated in response to the previously mentioned three-month ethanol treatment. Moreover, after chronic ethanol administration, hypermethylation was present both in the gene associated with folylpolyglutamate hydrolase and in vitamin B9 transporter genes [93]. This elucidated the fact that ethanol affects vitamin B9 absorption at the genomic level [93,94]. Regardless of studies showing genomic impact of ethanol on the vitamin B9 transporter, it is important to note that there are other studies reporting no changes in vitamin B9 absorption following treatment with ethanol. These discrepancies may result from a number of factors, including the experimental procedures, models, and even types of ethanol used [16,88].

#### 3.1.5. Vitamin C Absorption

Recent studies have demonstrated that ethanol directly affect the intestinal vitamin C co-transporter [95]. Kunming mice were administered with ethanol for two weeks with a diet comprising 25% ethanol. The protein expression of two intestinal sodium-dependent vitamin C co-transporters (SLC23A1–2) were significantly increased. However, the serum levels of ascorbate did not change. Vitamin C supplementation reversed the increase in expression of the vitamin C co-transporters in combination with decreased expression of hypoxia-inducible factor-α, suggesting a substrate-induced regulation of the transporter. Importantly, this study also demonstrates that ethanol inhibition of intestinal absorption may not apply to all nutrients [95].

#### 3.1.6. Vitamin B12 Absorption

Available research describing the effect of ethanol on vitamin B12 absorption is very limited. Vitamin B12 absorption is of critical importance because deficiency of this vitamin leads to macrocytic anemia [117]. In one study, rats fed a liquid ethanol diet composed of 35% ethanol displayed decreased vitamin B12 absorption, but this was not due to the binding of the vitamin B12 complex to the BBM receptors [40,110]. Further research is needed to understand the mechanistic details of the effect of ethanol on the absorption and availability of this vital nutrient.

Little is known on the effect of ethanol consumption on fat-soluble vitamins. Concentrations of fat-soluble vitamins are inhibited in chronic alcoholics, but the concentrations vary based on the individual [1]. More research is necessary on the mechanisms underlying this inhibition.

### 3.2. The Effect of Ethanol on Micronutrients—Minerals

Ethanol affecting the absorption of minerals along the small intestine, including calcium [9,96], zinc [97,98,99], iron [100,101], and magnesium [102], has been reported. Studies have shown that net calcium absorption was inhibited in rats given moderate (2 g/kg) ethanol, but the treatment also increased calcium secretion, leading to no net change in calcium serum levels [71,96]. So far, no alterations in magnesium absorption have been shown at the nutrient transporter level, but hypomagnesaemia is common in chronic alcoholics [1]. More research has focused on the effect of ethanol consumption on iron and zinc absorption.

#### 3.2.1. Iron Absorption

The interaction between ethanol and intestinal iron absorption is complex. Several studies have established that chronic alcohol consumers have high concentrations of iron due to its increased absorption. Ethanol’s effect on iron’s linear absorption is possibly due to its increase in passive absorption, which itself is due to increased intestinal permeability in response to ethanol [100]. In another study, duodenal iron absorption was unchanged following thirteen weeks of ethanol in male Swiss mice. While the duodenal iron absorption did not change, in the terminal small intestine, iron absorption was increased, suggesting regional variability of the effects of ethanol on iron absorption [101]. However, in 2016, a heavy eight- to twelve- week dosage of ethanol in male Swiss mice showed decreased duodenal protein expression of the iron transporters divalent-metal transporter-1 (DMT1; SLC11A2) and ferroportin 1 (FPN1; SLC40A1) [101,118]. Recent publications connected the downregulation of a key iron metabolism regulator, hepcidin, in mice following ethanol administration. More specifically, hepcidin 1, but not hepcidin 2, was downregulated. The downregulation of hepcidin corresponded to an increased duodenal protein expression of DMT1 and FPN1. Hepcidin has been shown to directly regulate both transporters’ expression patterns, with sex-specific differences [118]. Furthermore, the oxidative stress created by alcohol metabolism has been suggested to alter the C/EBPα transcription factor activity and subsequent downregulation of liver hepcidin and stimulation in iron transporter expression [119]. This study was supported by research conducted with duodenal samples from patients with alcoholic liver disease. These patients demonstrated increased mRNA expression of the RPN1 and DMT1 transporters [120].

Mechanistic studies on the impact of ethanol on intestinal iron transport demonstrated a complicated relationship between ethanol and iron absorption. Investigators demonstrated that supplementation of vitamin C in Kunming mice exposed to heavy ethanol in their drinking water for seven days decreased hepatic iron overload by decreasing intestinal ferroportin 1 and transferrin receptor 1 (TfR1) expression in the liver, leading to decreased blood iron levels [121]. Moreover, even exposure to a phytonutrient called Aplysin, purified from the red alga Laurencia tristicha, could slightly reverse both increased expression of the DMT1 and FPN1 and intestinal permeability in the small intestine caused by the ingestion of ethanol [122]. Clearly, further research is required for conclusive interference.

#### 3.2.2. Zinc and Selenium Absorption

Chronic alcohol use has been linked to ileal zinc deficiency [1,97]. However, it was not until very recently that this phenomenon was described in detail. Zinc transporter mRNA expressions were inhibited in the duodenum of male rodents exposed to ethanol via six-weeks of the Lieber DeCarli ethanol diet (SLC39A1; SLC39A4; SLC30A4) [55]. Furthermore, in pregnant rats exposed to ethanol, zinc was conserved in the mothers, and zinc absorption was increased in the offspring. Such conservation of zinc in the mothers suggests that the human body will adapt to ethanol-dependent nutrient deficiencies during pregnancy [123]. Extensive research has focused on the relationship between zinc and cellular junction regulation in the small intestine. Zinc plays a clear role in ethanol-mediated intestinal barrier disruption by decreasing the presence of cellular junction proteins, including claudin, ZO-1, and occluding [56]. This may be due to regulation in the ileum by hepatocyte nuclear factor-4α [56]. However, ethanol’s impact on the intestinal function and expression of zinc transporters requires further research. In the duodenum, selenomethionine absorption was increased following heavy ethanol dosages (20% *v*/*v*) in Wistar rat offspring for four weeks [104]. Exposure to heavy ethanol levels significantly increased the affinity of the transporter (Km). This is one of the few studies that focused on the impact of gestational and lactational ethanol treatment on intestinal nutrient absorption, and further research using this model system is necessary [104].

### 3.3. The Effect of Ethanol on Macronutrients—Fats

Several studies have described that ethanol could exert varied effects on lipid absorption along the small intestine. Moderate ethanol consumption can either stimulate or have no effect on dietary fat absorption. In one study, ethanol increased oleate absorption, but did not alter the overall long-chain fatty acid concentration in the portal pathway [124]. In another study, dietary fat absorption was increased in response to a moderate dosage of ethanol (0.75 g/kg per hour). At the same time, however, fat absorption was inhibited by chronic ethanol use. For example, three to four weeks of 36% ethanol inhibited dietary absorption of fats in rats [75]. Decreased absorption of fatty acid, arachidonic acid, and linoleic acid was reported in the jejunum of chronic alcoholics following heavy ethanol consumption (100 mM) [79,106]. In summary, the effect of ethanol on dietary fat absorption is dependent on ethanol dosage.

Dietary lipid composition also has been shown to affect glucose uptake in rats fed isocaloric diets high in saturated or polyunsaturated fats. Both diets prevented the jejunal uptake of glucose, galactose, medium and long-chain fatty acids, and cholesterol in rats fed 15% ethanol in their drinking water for four weeks [125]. Moreover, rats fed with a diet containing saturated fatty acids prevent the inhibitory effects of acute and chronic ethanol exposure on jejunal glucose uptake [125]. Beyond these studies, no work has investigated the effect of ethanol consumption on fat absorption, specifically cholesterol, bile acids, and triglycerides, along the small intestine. In all, ethanol affects the absorption of lipids along the small intestine. Moreover, the composition of lipids in diets are important to consider when examining the overall effect of ethanol consumption on nutrient absorption [125].

### 3.4. The Effect of Ethanol on Macronutrients—Carbohydrates

Ethanol consumption effects carbohydrate absorption in multiple ways [1,111]. Following a heavy dosage of ethanol, xylose absorption was inhibited [18], but fructose absorption was not altered in the small intestine [111]. However, these differ from ethanol’s effect on another carbohydrate: glucose.

#### Glucose Absorption

Glucose absorption is the main source of energy, and its absorption has been extensively investigated during the initial wave of ethanol and intestinal transporter research. Among the first to describe an inhibition in glucose absorption were Ghirardi and colleagues in 1971 [7]. However, it was Dinda and colleagues in 1975 that elucidated the impact of ethanol on glucose absorption [10]. Using a binge-dosage of ethanol equivalent to 450 mM in the jejunum of hamsters, investigators demonstrated that ethanol inhibited the active transport of glucose without a change in the net flux of sodium. Further experimentation, over many different studies and model systems, determined that ethanol decreased glucose absorption at the level of BBM in hamsters [10,14,47,65], rats [66,69,72,112], dogs [71], and more recently, chickens [73]. A heavy dosage of ethanol affects intestinal glucose absorption at the BBM of small intestinal cells via a reduction in the maximal rate of uptake, rather than by a change in the affinity of the transporter [72].

Previous publications have found the same inhibitory results using a more moderate dosage of ethanol [73]. In 2011, using an Ussing chamber in the jejunum of chickens, Yunus and colleagues found that a moderate dosage of ethanol inhibited intestinal glucose absorption [73]. The latest study, conducted in 2019 using a moderate dosage of ethanol, further described the mechanism behind ethanol’s inhibition of glucose absorption [74]. In the rat intestinal epithelial cell (IEC) line known as IEC-18 cells, a dosage of moderate ethanol caused an inhibition in glucose absorption through a decrease in the affinity of the transporter SGLT1 (SLC5A1) to glucose. However, there was no alteration in the maximal rate of uptake of SGLT1. This result was supported with unaltered protein expression levels of the SGLT1 co-transporter. The same results were then shown in an in vivo Sprague Dawley rat model using a single moderate dosage of ethanol. While this mechanism of action differed from what studies had previously established, that difference may have arisen from the dosage of ethanol used. Overall, further research is needed, both at varying ethanol dosages and at the molecular signaling level, to better understand ethanol’s impact on this vital sodium-glucose co-transporter [74].

### 3.5. Peptides and Amino Acids Absorption

Ethanol inhibits the absorption of amino acids, such as alanine [111], glycine [6], leucine [80,81,82,112], and glutamine. However, more research is necessary to clarify the reported null effect of low concentration of ethanol, below 200 mM, on glycine and leucine absorption [70], including more investigations of the mechanisms underlying these effects. Dipeptides and tripeptides are absorbed primarily in the proximal small intestine using proton-coupled oligopeptide transporters. One type of proton-coupled oligopeptide transporter is PepT1 (SLC15A1). Using Xenopus laevis oocytes, the human PepT1 was expressed and exposed to 200 mM ethanol for three minutes. While investigators found no change in the affinity of the co-transporter (Km), there was a significant inhibition of the currents for glycyl-sarcosine and alanyl-alanine [126]. This was the first study to investigate ethanol’s effect on the human PepT1 transporter.

#### Glutamine Absorption

In certain organs, glutamine is a vital nutrient, contributing to its designation as a conditionally essential amino acid in the small intestine. In the small intestine, glutamine is the main energy source for intestinal enterocytes. Glutamine is essential to the maintenance of the intestinal epithelium [127,128]. Only one recent study has investigated the absorption of this amino acid following ethanol treatment [33]. Utilizing a moderate dosage of ethanol, Sprague Dawley rats attained a blood alcohol content (BAC) of 0.04%, which significantly inhibited BBM absorption of glutamine. The decrease was due to an inhibition in the maximal rate of uptake of the co-transporter (Vmax). In fact, investigators demonstrated decreases in the protein expression of the primary glutamine co-transporter in the villus cells of the small intestine: B0AT1 (SLC6A19). B0AT1’s protein expression was decreased at both the whole cell homogenate and BBM of villus cells, a phenomenon that investigators also demonstrated in an in vitro model using IEC-18 cells. Taking this a step further, investigators concurrently showed that the mechanism of inhibition at a cellular level was based on the protein kinase C (PKC) pathway. Using IEC-18 cells and treatments with Calphostin C, a PKC inhibitor, investigators determined a PKC-dependent reversal of ethanol’s inhibitory action on glutamine absorption. Moreover, PKC-α knock-down studies showed the same reversal as well. Further research is required to better comprehend the intracellular mechanisms involved in this phenomenon [33].

## 4. Conclusions and Future Perspectives

In recent years, the field of alcohol consumption and its effect on intestinal nutrient absorption at the BBM of small intestine has continued to expand. Mounting studies reported ethanol effect on the absorption of array of macro and micronutrients, including glucose [73,74], glutamine [33] vitamin B2 [90], vitamin C [95], vitamin B1 [88,107], vitamin B9 [92,94,109,129], iron [101,118,119,120,121,122], zinc [55,123], and selenium [104]. Additionally, further research has focused on the molecular mechanism of ethanol’s action on nutrient absorption, more particularly on the nutrient transporter. Dose response studies investigating the effect of mild, moderate, and heavy alcohol consumption on nutrient absorption will be important to precisely define the beneficial and detrimental role of ethanol. Most Americans have consumed alcohol at a moderate level in the past month, according to the NIAAA [27,28,130]. Nevertheless, only few studies in the literature focus on long term moderate alcohol consumption and its effect on intestinal nutrient absorption. Another significant gap in the field of ethanol consumption and nutrient absorption is the lack of sex-specific data, as well as ethanol’s effect on nutrient absorption during maternal gestation and lactation [123]. Additionally, understudied are the potential differences in the effects of different type of alcohol, varying from liquor to wine on nutrient absorption.

Recent studies have shown that the absorption of nutrients, such as glucose, glutamine, and bile-acids, are stimulated during obesity [131,132,133,134]. Therefore, studies focused on the effect of ethanol consumption in ameliorating or aggravating altered nutrient absorption in various disease state, such as obesity (characterized by increase nutrient absorption) and inflammatory bowel disease (characterized by decrease nutrient absorption), are also likely to define nice therapeutic strategies [131,132,133,134,135]. Mechanistic studies on the effect of ethanol on nutrient transporters could be better achieved by utilizing the recently developed ex vivo model of human intestinal organoid culture [136,137,138,139]. The sustained culture of human intestinal organoids, three-dimensional cell cultures derived from human biopsy tissues, can be used to study the effects of ethanol on nutrient absorption [140]. Recently, this model has been utilized in studies involving intestinal alcohol-injury [141]. Moreover, this model system proves to be advantageous, as biopsies can be derived from patients with different disease characteristics, including obese and IBD patients [142,143]. Overall, additional extensive research is required to fully comprehend the intricate balance of nutrient homeostasis and the impact of ethanol consumption on this malleable system.

## Figures and Tables

**Table 1:** The Effect of Ethanol on Macronutrient Intestinal Nutrient Absorption.

Nutrient	Effect on Absorption	Dosage of Ethanol	Location	References
Amino Acids	↓	Heavy Binge	SI	[6,105]
Peptides	↓, n/c *	Heavy	Duodenum	[78,79]
Lipids	↓, n/c *	Heavy	Duodenum SI	[68,75,77,78,79,106]
Water	↓	Heavy	Jejunum	[15,17]

Adapted with permission from Butts M.R. [32]. Abbreviations: *, n/c only present in upper jejunum; ↓, decrease; heavy, blood alcohol content (BAC) ≥ 0.08%; binge, BAC ≥ 0.08% in less than two hours; SI, small intestine with no specified location.

**Table 2:** Summary of the Main Studies on the Effect of Ethanol on Intestinal Nutrient Absorption.

Nutrient	Effect on Absorption	Dosage	Location	References
Vitamin B1	↓ or n/c *	Heavy Binge	Jejunum, HuTu-80 cells	[11,83,84,85,86,87,88,89]
Vitamin B2	↓	Heavy	Jejunum, Colon	[90]
Vitamin B7	↓	Heavy	SI, Colon	[107,108]
Vitamin B9	↓, n/c *	Heavy Binge	Jejunum	[8,16,91,92,93,94,109]
Vitamins A, D, E, B6, B12, and K	↓	Heavy	SI	[1,13,110]
Vitamin C	↑	Heavy	SI	[95]
Glucose	↓ or n/c **^,#,##^	Heavy Binge Moderate	SI, Jejunum, IEC-18 cells	[7,10,14,17,65,66,67,68,69,70,71,72,73,74,111,112]
Fructose	n/c	Heavy	SI	[111]
Glutamine	↓	Moderate	IEC-18 cells, Terminal SI	[33]
Leucine	↓ or n/c **	Heavy	Jejunum	[70,72,80,81,82]
Glycine	↓ or n/c **	Heavy	SI	[6,70]
Xylose, alanine ^#^, ascorbate ^#^	↓	Heavy	SI	[18,111]
Zinc	↓	Heavy	SI	[98]
Calcium	↓	Heavy, Moderate	Duodenum	[9,96]
Iron	↑ and n/c ^###^	Heavy	SI	[100,101]
Magnesium	↓	Heavy	SI	[17]
Selenium	↑	Heavy	Duodenum	[104]

Adapted with permission from Butts M.R. [32]. Abbreviations: *, n/c, no change, only refers to Lemos et al. (2005); **, n/c only present in concentration below 200 mM from Hwang et al. (1989); ^#^, only refers to O’Neill et al. (1986), and less than 1% for glucose; ^##^, only refers to Ghirardi et al. (1971) in the 20 day treatment; n/c, no change; ^###^, increase in duodenum, n/c in ileum; ↓, decrease; ↑, increase; heavy, BAC ≥ 0.08%; moderate, BAC ≤ 0.04%; binge, BAC ≥ 0.08% in less than two hours; SI, small intestine with no specified location; IEC, intestinal epithelial cells.

## Data Availability

Not applicable.
